# A prognostic index that predicts outcome following palliative whole brain radiotherapy for patients with metastatic malignant melanoma

**DOI:** 10.1038/sj.bjc.6602018

**Published:** 2004-08-10

**Authors:** S L Morris, S H Low, R P A'Hern, T G Eisen, M E Gore, C M Nutting, K J Harrington

**Affiliations:** 1 Melanoma Unit, The Royal Marsden Hospital NHS Trust, 203 Fulham Road, London SW3 6JJ, UK; 2 Institute of Cancer Research, 237 Fulham Road, London SW3 6JB, UK

**Keywords:** melanoma, palliative radiotherapy, brain metastases, prognosis, prognostic index

## Abstract

To determine the outcome of patients with metastatic malignant melanoma (MMM) treated with palliative whole brain radiotherapy (WBRT) and to identify factors that predict treatment outcome to assist future trial design, a retrospective study was performed on patients with MMM who received WBRT at the Royal Marsden Hospital between 1998 and 2003. Data regarding patient factors, tumour factors and survival were collected. A total of 112 patients were identified and full data were available for 102 patients. The median age was 53 years (range 25–81 years), 66.7% were male and 33.3% female. The median dose prescribed was 20 Gy in five fractions as a mid-plane dose. The median survival after WBRT for the whole group was 51 days (range 3–1386). In an attempt to define prognostic groups, we used the validated RTOG recursive partitioning analysis (RPA) classification for brain metastasis (class 1: Karnofsky Performance Score (KPS) ⩾70%, age <65 years with no extracranial metastasis; class 3: KPS <70%; class 2: all others). The median survivals were 151, 71 and 21 days for RPA class 1, 2 and 3, respectively (*P*<0.001). Multivariate analysis showed that RPA class, leptomeningeal involvement, presence and number of extracranial metastatic sites and progressive disease in the brain on imaging before WBRT are important independent predictive factors. A prognostic index was derived from these factors that allowed identification of patients unlikely to benefit from WBRT. In conclusion, the RTOG RPA classification is valid when applied to patients with MMM. Patients in RPA class 1 and good prognosis class 2 are likely to benefit from palliative WBRT and should be considered for entry into trials that aim to improve duration of response. We identified that patients with RPA class 3, leptomeningeal involvement or RPA class 2 with poor prognostic index are unlikely to benefit from palliative WBRT.

Brain metastases are a frequent development in the natural history of metastatic malignant melanoma (MMM). They present clinically in 8–46% of patients and are found at autopsy in 55–75% (Amer *et al*, 1978; Patel *et al*, 1978; Bullard *et al*, 1981). The prognosis is measured in months and the aim of treatment is palliative. The optimum role of surgery, stereotactic radiotherapy (SRT) and whole brain radiotherapy (WBRT) remains undefined and controversial.

Surgery and SRT may be used when disease is limited to less than three lesions. Many studies show improved survival in those undergoing resection and also in those with no extracranial metastases (Katz 1981; Madajewicz *et al*, 1984; Choi *et al*, 1985; Ziegler and Cooper 1986; Skibber *et al*, 1996; Ellerhorst *et al*, 2001). A series of 91 patients undergoing surgical resection of MMM brain metastases showed that the outcome was better if the patient had no neurological deficit, a single supratentorial site and no lung or visceral metastases. It also showed no benefit to adding WBRT with a median survival of 9.5 months with WBRT and 8.3 months without (Wronski and Arbit, 2000). A study of 122 patients treated with gamma knife radiosurgery showed a median survival of 7 months with improved survival and freedom from tumour progression in those with tumours <3 cm and inactive systemic disease. It also showed no benefit of WBRT in multivariate analysis (Yu *et al*, 2002). It must be remembered that the patients selected for surgery or SRT are those with a low disease burden and good performance status who are likely to benefit from a more favourable natural history of their malignancy.

Whole brain radiotherapy is commonly used for palliation for patients with large or multiple metastases. It has been shown to provide symptomatic improvement in 76% of patients (Carella *et al*, 1980). It is most commonly combined with corticosteroid therapy, which is discontinued on completion of WBRT and resolution of symptoms. The largest reported series of WBRT in MMM is by Sampson *et al* (1998) who treated 180 patients with a median survival of 120 days. Other smaller series report similar median survivals in the range of 9–20 weeks (Madajewicz *et al*, 1984; Choi *et al*, 1985; Ziegler and Cooper, 1986; Barth *et al*, 1995; Gupta *et al*, 1997; Ellerhorst *et al*, 2001). Numerous studies of the effects of fraction size on palliation and survival have failed to demonstrate a survival advantage and many authors recommend a total dose of at least 30 Gy (Ellerhorst *et al*, 2001). We currently recommend a dose of 20 Gy in five fractions over 1 week.

The role of systemic agents with the ability to cross the blood–brain barrier has been investigated. Drugs such as fotemustine and temozolomide have reported single-agent response rates of 25% (Douglas and Margolin, 2002). They are currently being studied in combination with WBRT. A study of temozolomide with WBRT for MMM in 31 patients has shown a median survival of 6 months, a CR in only one patient and PR in only two patients leading the authors to conclude that WBRT has a lower than expected activity (Margolin *et al*, 2002). A recent French randomised phase III trial in 76 patients showed that fotemustine plus WBRT delayed the time to brain tumour progression compared to fotemustine alone, but without significant improvement in terms of objective control or overall survival (Mornex *et al*, 2003).

As a background to designing future studies of palliative WBRT in patients with brain metastases from MMM, we have conducted a retrospective review of patients treated at the Royal Marsden Hospital in the last 5 years. The focus of this work was an attempt to define patient- and disease-related characteristics predictive of treatment outcome. As a starting point for this analysis, the RTOG recursive partitioning analysis (RPA) classification of brain metastasis that has been validated in lung and breast cancer patients (Gasper *et al*, 2000) was applied to this group of patients.

## PATIENTS AND METHODS

The Royal Marsden Hospital database was searched for patients with MMM who received WBRT between January 1998 and June 2003. A total of 112 patients were identified and their case notes and radiotherapy records were reviewed. Data were collected regarding patient factors, tumour factors, treatment and survival. We contacted the general practitioners of patients lost to follow-up to obtain further information.

Information was collected on the patient demographics, date of diagnosis of melanoma, date of diagnosis of brain metastases (taken as the date of the diagnostic CT or MRI scan), local and systemic disease status, date of completion of WBRT and the date of death or last follow-up. The cause of death was recorded if available. The radiotherapy dose and schedule were recorded. The indications for WBRT were stratified as follows: symptoms (headache, nausea and vomiting), fits, neurological deficit, postoperative radiotherapy, asymptomatic progression and progressive disease on imaging (carried out to investigate progressive neurological symptoms or as part of a staging scan to assess response to systemic therapy). The number of brain metastases and the presence or absence of leptomeningeal involvement on imaging were recorded. From the notes and imaging reports, we recorded the presence and sites of extracranial metastases and if the disease was stable or progressing. Any response in the brain following WBRT on imaging was noted, although this parameter was only assessed in a small number of patients. The details of all prior chemotherapy, surgery and SRT were recorded.

Because of the retrospective nature of this study, no data were available on the palliative benefit of WBRT. The symptomatic benefit of WBRT has been studied, and separating the relative benefits of steroids and radiotherapy is difficult. If patients survive long enough, they will obtain a symptomatic benefit from WBRT in up to 76% of cases (Carella *et al*, 1980). Our aim with this review was to identify factors predicting a poor outcome and to help identify those who are unlikely to survive long enough to see a palliative benefit.

To use the RTOG RPA classification data on age, extracranial metastasis and Karnofsky Performance Status (KPS) were needed (Gasper *et al*, 2000). We then assigned RPA class 1 to those with KPS ⩾70%, age less than 65 years with controlled primary and no extracranial metastasis, RPA class 3 to those with KPS <70% and RPA class 2 to all others (i.e. KPS ⩾70%, age ⩾65 years, uncontrolled primary or evidence of extracranial metastases). The KPS was not formally recorded prospectively in the majority of cases, but it was relatively easy to determine from the detailed case note entries prior to commencement of WBRT if the KPS was ⩾70 or <70%. In cases with insufficient information in the case notes, an RPA class was not assigned.

### Radiotherapy technique and delivery

Whole brain radiotherapy was delivered to all patients using a thermoplastic or Perspex immobilisation shell. All the brain tissue above a baseline extending from the supraorbital ridge to the external auditory meatus was treated with parallel-opposed lateral fields with the dose prescribed at the mid-plane. Treatment was delivered using a ^60^Co source or a 6 MV linear accelerator in all cases.

### Statistical analysis

The median survival after WBRT was calculated from the date of completion of radiotherapy to the date of death or the date of last follow-up in those still alive at the time of analysis. The Kaplan–Meier method was used to plot survival curves and the log-rank test was used to analyse differences between groups. Data analysis was performed using an SPSS statistics program. Univariate and multivariate analyses were performed and a prognostic index was formulated.

## RESULTS

### Patient characteristics

In the time period of the study, 112 patients were treated with WBRT for MMM. Complete survival and follow-up data were available on 102 patients. We excluded 10 patients for whom we could not obtain follow-up details. The data analysed were incomplete in 23 patients. Of these, data were incomplete on extracranial metastases in six, on the number of brain metastases in four, on whether the brain metastasis has progressed on CT imaging in 20 and in four patients it was not possible to assign an RPA class due to insufficient information.

The median age of the patients was 53 years (range 25–81 years), 68 were male and 34 female. The median time from diagnosis of melanoma to diagnosis of brain metastasis was 976 days (range 0–4808 days). Four patients had brain metastasis at presentation, and 77 patients had extracranial disease that was stable in 38 patients and progressive in 39 patients. The number and sites of extracranial metastases were recorded and are shown in Table 1

**Table 1:** Patient characteristics

	**Number**
Gender	M=68, F=34
Age (years)	53 (median)
Indications for WBRT	
Symptoms	72
Fits	3
Neurology	41
Postoperative	7
Asymptomatic PD in brain	2
PD in brain on imaging	28
Extra-cranial metastasis	77
Stable disease	38
Progressive disease	39
Number of metastatic sites	
1	20
2	28
3	15
4	8
5	4
NK	2
Site of metastasis	
Liver	24
Lung	52
Lymph nodes	50
Soft tissue/subcutaneous	18
Spleen	9
Adrenal	12
Other	18
Number of brain metastasis	
1	18
2	15
Multiple (⩾3)	61
NK	8
RPA class	
1	14
2	54
3	30
NK	4

. In all, 18 patients had a single brain metastasis, 15 two metastases and 61 multiple (⩾3) metastases. The indications for WBRT were symptoms in 72 patients, fits in three patients, neurological deficit in 41 patients, postoperative radiotherapy in seven patients and progressive disease (PD) in the brain on imaging (CT or MRI) in 28 patients. Only two patients receiving WBRT had asymptomatic progressive intracranial disease.

### Treatment

All 102 patients received WBRT at the Royal Marsden Hospital with either a ^60^Co teletherapy source or a 6 MV linear accelerator. A range of doses was used as follows: 20 Gy in five fractions – 76 patients; 30 Gy in 10 fractions – seven patients; 24 Gy in six fractions – five patients; 30 Gy in six fractions – three patients; other fraction schedules – three patients; failed to complete radiotherapy – eight patients.

Seven patients were treated with radiotherapy postoperatively and five patients had had prior surgery for brain metastases with WBRT given at the time of disease progression. Two patients had received prior SRT and four patients had SRT after WBRT on progression. In all, 46 patients had received prior chemotherapy (one regimen (35 patients), two regimens (seven patients), three regimens (two patients), four regimens (two patients)), 15 patients received initial chemotherapy (fotemustine (seven patients), temozolomide (five patients), DTIC (one patient), interferon-*α* (one patient), a phase 1 drug (one patient)) for brain metastasis with WBRT delivered at the time of progression, and 12 patients had received prior adjuvant interferon-*α*.

### Survival

At the time of data analysis, five patients were still alive. The cause of death was recorded as metastatic melanoma in 71 patients and cerebrovascular accident in one patient. We were unable to determine if death was due to intracerebral or extracranial disease in all but a few patients. The certified cause of death was unknown in 25 patients.

Of the 102 patients, 24 had CT imaging to assess response after WBRT. Response data were recorded according to the WHO criteria. Four patients had evidence of radiological partial response and eight patients had a minor response (<50% reduction). One patient had stable disease and 11 had evidence of disease progression.

**Figure 1:**
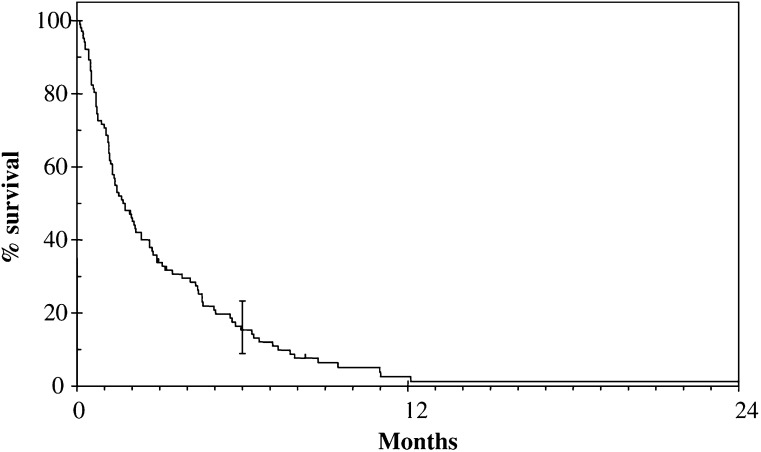
Overall survival of 102 patients with cerebral metastases from malignant melanoma.

**Figure 2:**
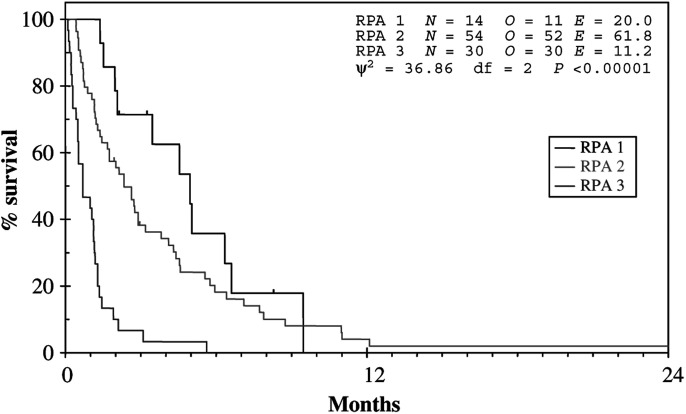
Survival according to RPA class.

The median survival after WBRT for the whole group was 51 days (95% CI 34–68, range 3–1386) (see Figure 1

). Using the RTOG RPA classification for brain metastases, there was a significant difference in survival between the classes (see Figure 2

). Class 1 had a median survival of 151 days (95% CI 78–224, range 42–288), class 2 a median survival of 71 days (95% CI 45–97, range 13–1386) and class 3 a median survival of 21 days (95% CI 2–40, range 3–171) (*P*<0.001). Patients with no extracranial metastases had a median survival of 105 days (range 4–335 days) and those with extracranial metastases had a median survival of 39 days (range 3–1386 days) (*P*=0.016). In patients with extracranial metastases, those with stable disease (*n*=38) survived a median 40 days (range 13–1386 days) and those with progressive disease (*n*=39) survived a median 39 days (range 8–368 days). Seven patients had leptomeningeal involvement on imaging and had a median survival post-WBRT of 23 days (range 3–63 days) (*P*=0.003). Eight patients did not complete the prescribed course of WBRT due to clinical deterioration and had a median survival of 7 days (range 3–9 days).

The results of univariate and multivariate analyses are shown in Tables 2

**Table 2:** Univariate analysis

	**No.**	**HR (95% CI)**	***P*-value**
RPA class (*n*=98)			
1	14	1	
2	54	1.61 (0.84–3.09)	
3	30	5.93 (2.88–12.21)	<0.001
Time from diagnosis to brain metastases (*n*=95) (years)			
<1.5	25	1	
1.5–3	26	1.24 (0.71–2.19)	
>3	44	0.84 (0.51–1.39)	0.31
PD in brain on imaging prior to WBRT (*n*=92)			
No	54	1	
Yes	28	1.06 (0.65–1.71)	0.82
Meningeal disease (*n*=95)			
No	88	1	
Yes	7	3.13 (1.41–6.95)	0.005
Number of brain metastases (*n*=94)			
1	18	1	
2	15	0.97 (0.48–1.97)	
⩾3	61	1.29 (0.74–2.25)	0.48
Number of ECM sites (*n*=96)			
0	19	1	
1	20	1.38 (0.71–2.67)	
2	29	2.2 (1.18–4.09)	
3	16	2.11 (1.05–4.23)	
4+	12	5.89 (2.68–12.94)	<0.001
ECM and response (*n*=96)			
None	19	1	
SD	38	1.5 (0.91–2.66)	
PD	39	2.31 (1.35–3.96)	0.009
Prior chemotherapy (*n*=93)			
No	47	1	
Yes	46	1.32 (0.87–2.0)	0.2

Data were collected on 102 patients, but in all categories some data were missing. The number of patients for whom data were available in each category is indicated in parentheses (e.g. RPA class (*n*=98)).

and 3

**Table 3:** Multivariate analysis

	**No.**	**HR (95% CI)**	***P*-value**
RPA class			
1	13	1	
2	42	1.31 (0.56–3.06)	
3	26	3.78 (1.28–11.16)	0.004
PD in brain on imaging prior to WBRT			
No	54	1	
Yes	27	2.06 (1.19–3.59)	0.01
Meningeal disease			
None	75	1	
Yes	6	3.31 (1.27–8.61)	0.014
Number of ECM sites			
0	18	1	
1	16	0.68 (0.23–2.0)	
2	22	1.63 (0.57–4.64)	
3	14	1.37 (0.46–4.12)	
4+	11	4.13 (1.18–14.42)	0.012

Full data were available on all parameters for 81 patients for the purpose of multivariate analysis.

, respectively. The multivariate model implies that RPA, meningeal involvement, number of extracranial metastatic sites and progressive disease in the brain on imaging are important independent predictive factors. A prognostic index summarising the contribution of each of these factors to prognosis was calculated by creating a single score summarising the relative contribution of each factor (Table 4

**Table 4:** Prognostic index^a^

**Score**	**Number**	**HR**	**Median survival (days)**
2–4	17	1	138
5–6	16	1.2	80
7–8	24	2.2	42
9–10	14	9.6	18
11+	10	14	15

a Prognostic index=number of ECM sites+(2 × RPA)+(2 if PD in brain on imaging)+(4 if meningeal).

). The prognostic score was calculated using the formula prognostic score=(number of ECM sites+(2 × RPA)+(2 if PD in brain on imaging)+(4 if leptomeningeal involvement)).

The resulting index was then divided into approximately equal-sized groups and its performance examined. Prognostic indices inevitably perform well if they are both derived and tested on the same set of data, so strictly this index still requires external validation. However, taking as the starting point that RPA class 1 patients should be treated and those that are class 3 should not be treated, the index enables the intermediate group (class 2) to be split into those that should and should not be treated. If the score number of other sites+2 (if progressive disease in brain on imaging)+4 (if leptomeningeal involvement) is 5 or greater, then patients would only be expected to have a median survival of 21 days or less.

It is noteworthy that progressive disease on imaging before starting WBRT was a significant prognostic variable on multivariate, but not on univariate, analysis. This apparent anomaly is probably accounted for by the fact that showing progressive disease on a CT or MRI scan is inversely correlated with RPA, the most highly predictive single factor. The proportions showing progressive disease on imaging prior to WBRT in the three RPA groups were 54% (class 1, *n*=13), 40% (class 2, *n*=43) and 15% (class 3, *n*=22). The relatively high proportion of good prognosis patients with progressive disease on imaging prior to WBRT is likely to be the reason as to why it was not significant on univariate analysis. However, within the RPA classes, the patients with progressive disease on imaging prior to WBRT have a worse outcome. For example, in a Cox model with RPA and progressive disease on imaging prior to WBRT as factors, the latter has a hazard ratio of 2.2 (*P*=0.01).

For the patients who had a documented partial or minor response on CT imaging after WBRT, three patients were in RPA class 1 and nine were in RPA class 2. Nine of the patients received radiation doses of 20 Gy in five fractions. The median prognostic index scores were 6.5 (range 5–7) for those with a partial response and 4.5 (range 2–7) for those with a minor response.

## DISCUSSION

This retrospective review confirms the data from other studies that patients with brain metastases from MMM have a very poor prognosis. The median overall survival of approximately 7 weeks is similar to that quoted by other authors who have reported median survivals of between 9 and 20 weeks (Madajewicz *et al*, 1984; Choi *et al*, 1985; Ziegler and Cooper, 1986; Barth *et al*, 1995; Gupta *et al*, 1997; Ellerhorst *et al*, 2001). Clearly, in a situation with such a poor prognosis, it is important to attempt to deliver treatment according to rational guidelines based on the likelihood of gaining a palliative benefit. Ideally, patients with little or no prospect of clinical benefit could be identified and offered best supportive care and those with a better prognosis could be offered active treatment.

**Figure 3:**
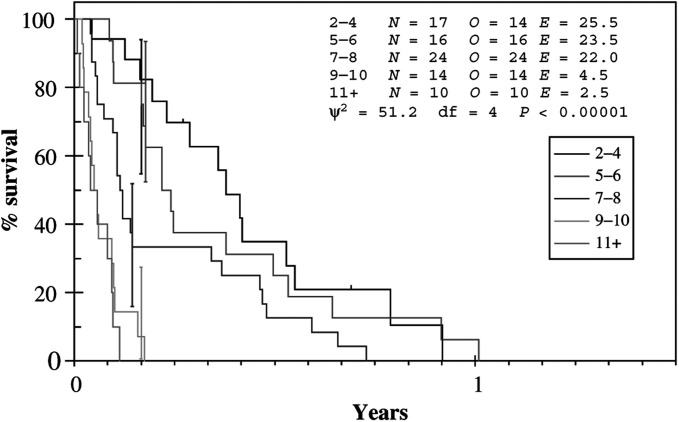
Survival by prognostic group.

To this end, the data presented here have shown that the RTOG RPA classification is valid when applied to MMM and facilitates the effective separation of patients into different prognostic groups (Figure 3

). In particular, this approach is successful in identifying patients with a very poor outcome following WBRT. Patients in RPA class 3 and those with leptomeningeal disease were found to have the worst prognosis with a median survival of only 3 weeks. This group of patients should not receive palliative WBRT but, rather, should be treated with best supportive care alone. Similarly, the presence of extracranial metastases was also defined as a poor prognostic factor with a median survival of 39 days. Interestingly, there was no difference in median survival if the extracranial metastases were deemed stable or progressive.

The data clearly demonstrate that patients in RPA class 1 and 2 should be considered for active treatment. Those in class 1 are most likely to benefit from aggressive management and should be considered for surgery, SRT, WBRT or cytotoxic chemotherapy (or combinations of these modalities), depending on the clinical picture. Now that this group of patients can be identified, it will be important to design randomised clinical trials to determine the optimal treatment approach for them. However, a greater challenge lies in identifying those patients in class 2 who are likely to benefit from WBRT and those who are not. By applying the derived prognostic index, it can be seen that those in RPA class 2 with a score of 9 or greater (i.e. leptomeningeal disease, five ECM sites or three ECM sites and PD in brain on imaging) have a median survival of only 21 days and are unlikely to benefit from WBRT. Therefore, future clinical trials in patients with brain metastases from MMM should direct patients in RPA class 2 with a prognostic score of 9 or more into studies of best supportive care. Patients with RPA class 2 and a prognostic score less than 9 should be considered for studies of WBRT with or without the addition of cytotoxic chemotherapy or biological agents.

These data provide important insights into the outcome of patients with brain metastases of MMM and demonstrate that it is possible to predict therapeutic outcome and to design future clinical studies on the basis of a number of simple clinical parameters. In the first instance, the RTOG RPA classification acts as an effective initial screen for patients who are likely to succumb rapidly to their disease. Therefore, such patients (RPA class 3 and 2 with a poor prognostic score) can be spared the unnecessary side effects of ineffective palliative WBRT and offered best supportive care alone. In contrast, patients in RPA class 1 and 2 with a better prognostic score can be considered for active therapeutic studies. By selecting and stratifying patients in this way, it should be possible to examine the role of palliative treatments in a more meaningful fashion than is currently possible.
